# Depression classification based on automatic ontology generation and natural language processing

**DOI:** 10.3389/fpsyg.2026.1780802

**Published:** 2026-04-02

**Authors:** Nicolae Goga, Andrei Vasilăteanu, Ramona Cristina Popa, Alexandru-Filip Popovici, Ramona Popovici, Maria Goga, Diana Todea, Alexandra Eni

**Affiliations:** 1 Department of Engineering in Foreign Languages, National University of Science and Technology Politehnica Bucharest, Bucharest, Romania; 2 Faculty of Psychology and Educational Sciences, University of Bucharest, Bucharest, Romania; 3 Doctoral School of Psychology and Educational Sciences, University of Bucharest, Bucharest, Romania; 4 Department of Training for the Teaching Career and Social-Humanities Sciences, National University of Science and Technology Politehnica Bucharest, Bucharest, Romania; 5 Department for the Training of Teaching Staff, Technical University of Civil Engineering of Bucharest, Bucharest, Romania; 6 Interdisciplinary School of Doctoral Studies, University of Bucharest, Bucharest, Romania; 7 Department of Psychology and Cognitive Sciences, University of Bucharest, Bucharest, Romania

**Keywords:** depression detection, digital health, natural language processing, ontology generation, religious communities

## Abstract

**Introduction:**

Depression is one of the most common mental disorders and one that has a great potential to affect people mentally, physically, and socially. Unfortunately, a majority of people either do not have access to treatment or avoid seeking help. In this context, many platforms have emerged to provide a space for discussion and support where users can interact anonymously.

**Methods:**

This study presents the results of our research on classifying depression in a specific community of religious people by analyzing texts posted on social media using semantic techniques, such as a comparative analysis of texts from users using ontologies. A supporting objective of the research was to create a natural language processing tool for classifying depression to obtain the corpus necessary for ontology creation.

**Results:**

The resulting ontologies were analyzed and compared with each other and also with existing ontologies in the literature on general depression.

**Discussion:**

The comparison was both qualitative and quantitative, taking into consideration similarity ratios for the quantitative comparison.

## 1 Introduction

Depression is among the most common mental disorders that has the potential to generate numerous psychological and physical problems, and it is the leading cause of disability worldwide [Bucci et al., 2019; Goodwin, 2006; World Health Organization (WHO), 2022]. In this context, research suggests that belonging to a religious community can be beneficial for mental health (Garssen et al., 2021; Keating, 2013; Manoiu et al., 2023; Nimrod, 2013). Religion can act as a coping mechanism by providing access to a community of people who share common values and beliefs, as well as through the messages it promotes (Kreski et al., 2022). Numerous studies have shown that religious involvement can reduce symptoms associated with depression by providing meaning and support from local communities (Bonelli et al., 2012; Kreski et al., 2022).

However, in some religious communities, there may be a stigma associated with various disorders, such as depression (Peteet, 2019). A lack of faith or engagement in behaviors considered sinful, which are not aligned with community norms, may contribute to more intense feelings of depression and may delay help-seeking (Peteet, 2019). Unfortunately, a majority of people with depression have limited opportunities to access specialized help. In this sense, one option is to turn to online communities, where anonymity is maintained, and individuals can monitor themselves and seek information (Goodwin, 2006; Naslund et al., 2020). One such platform is Reddit, a social platform that allows users to post topics anonymously (Boettcher, 2021; Guntuku et al., 2017). Recently, the platform has gained popularity as a source of public data, partly because of the distinctive ways it facilitates research (Boettcher, 2021).

On this platform, online interaction is facilitated by the fact that users can remain anonymous, which may be an essential aspect for some individuals (Highton-Williamson et al., 2015; Naslund et al., 2020; Nimrod, 2013; Okun and Nimrod, 2020). Compared to traditional self-assessment methods, data from social platforms offer the possibility of in-the-moment measurement of what a person is feeling and thinking (Guntuku et al., 2017). Recent studies suggest that it may be possible to identify some signs or symptoms of depression by analyzing user posts (Burdisso et al., 2021; Pirina and Çöltekin, 2018; Ren et al., 2021). In this context, there is a growing interest in utilizing ontology comparison as an investigative tool (Dragoni et al., 2018; Kontopoulos et al., 2013). Ontologies provide a way of organizing knowledge consisting of key concepts within a domain and the relationships between them, thereby capturing deep meaning that is relevant for classification purposes. Ontology-based analysis is particularly suitable for studying psychological constructs, such as depression, because it enables the structured semantic representation of symptoms, emotional states, cognitive patterns, and experiential concepts. Depression is a complex and multidimensional condition that manifests through language, such as expressions of mood, self-perception, social relationships, and behavioral tendencies. Ontologies allow these semantic elements to be systematically extracted, organized, and analyzed.

Previous research has demonstrated the effectiveness of ontology-based approaches in mental health applications, such as depression diagnosis support systems (Chang et al., 2015), social media analysis of depressive symptoms (Jung et al., 2015), and semantic-based sentiment and emotion analysis (Dragoni et al., 2018). These approaches show that ontologies can capture meaningful semantic patterns relevant to psychological states, making them a suitable tool for analyzing depression-related textual data.

In recent times, researchers use ontologies as a convenient way to store and share information (Chatterjee et al., 2023; Goga et al., 2019; Popa et al., 2017, 2018, 2019; Rubio et al., 2024). Ontologies can be written in various knowledge-representation languages, such as OWL/RDF, which can be parsed by computers while remaining understandable to researchers (Popa et al., 2019; Rubio et al., 2024; Ramona-Cristina et al., 2016). Several studies have addressed the automatic generation of ontologies, proposing different approaches and tools for this purpose (Al-Aswadi et al., 2020; Cross and Bathija, 2010; El Idrissi et al., 2013; Maedche et al., 2003; Kaushik and Chatterjee, 2016).

The majority of the existing studies, such as those mentioned above, rely on semi-automatic generation processes, require additional human review, or are difficult to apply across different domains, rather than employing fully automatic and user-friendly methods. In our research, we focus on the automatic generation of ontologies using the Text2Onto tool, a fully automated tool for the generation of ontologies that has been successfully applied in various research contexts (Cimiano and Völker, 2005).

Moreover, recent research has focused on using Natural Language Processing (NLP) techniques in broader tasks, where text processing and understanding can be used as tools for automatic diagnosis, the prediction of medical conditions, or the identification of potentially dangerous behaviors (Burdisso et al., 2019; Dowlagar and Mamidi, 2022; Hegde et al., 2022; Karmen et al., 2015; Kaushik and Chatterjee, 2016; Poświata and Perełkiewicz, 2022; Tausczik and Pennebaker, 2010; Trotzek et al., 2020; Yao et al., 2021; Ziemer and Korkmaz, 2017; Rosa et al., 2019).

In this article, we extend this line of research by applying this method, one of the first of its kind, to the classification of depression within a specific community of Christians. Another aspect of our study involves the use of automatic ontology generation on a corpus of social media posts to extract and compare concepts from texts written by depressed Christians, non-depressed Christians, and depressed individuals who are not necessarily Christians and non-depressed individuals who are not necessarily Christians, using both qualitative and quantitative approaches.

Despite considerable research being conducted on social media use in the general population, with several conclusions drawn regarding social support for individuals struggling with depression (Lu et al., 2021), few, if any, studies have focused on specific communities with particular traits that may influence how depression is experienced. One of the objectives of our study was to analyze the manifestation of depression in social network interactions, with a particular focus on religious communities.

As ontologies are difficult to create, a more feasible approach is to automate their generation from unstructured text (Elnagar et al., 2022). To date, automated ontology generation has neither been applied to texts authored by religious individuals with depression, nor has a comparative analysis been conducted between concepts extracted from texts written by religious individuals with depression and those written by non-religious individuals diagnosed with depression. Since ontology generation requires large amounts of text, another supporting objective was to create a tool for classifying posts as belonging to the depressed or control groups.

Research has shown that one way to classify users with depression is based on texts in which they explicitly say that they have been diagnosed by a physician (De Choudhury et al., 2021; Pedersen, 2015). Since not all Christian users provided such explicit statements, a natural language processing (NLP)-based AI engine was developed to classify these texts.

Engine training was based on posts from forums in which users explicitly stated that they had been diagnosed with depression by a physician. After training, the engine was applied to posts from forums where users did not mention their diagnosis in order to classify depression among Christian and non-Christian individuals.

The resulting classified texts were used to generate separate ontologies using an existing automatic ontology-generation engine (Text2Onto). The resulting ontologies were then analyzed and compared with existing ontologies reported in the literature on individuals with depression. The comparison was conducted both qualitatively and quantitatively, with similarity ratios used for quantitative comparison.

## 2 Materials and methods

### 2.1 Text selection and manual classification

Our study aims to analyze differences in social network posts across four user categories (depressed Christians, general Christians, general depressed individuals who are not necessarily Christians, non-depressed individuals who are not necessarily Christians). First, we created a corpus of texts based on searches conducted on Reddit and its subreddits, along with public forums and social media groups dedicated to depression-related topics. The selected texts contained direct, first-person references to depression (Bernard et al., 2016; Jarrold et al., 2011). Users identified themselves as belonging to a Christian denomination (orthodox, catholic, protestant, or neo-protestant).

Two main criteria were used to select the texts: the user’s report of a depression diagnosis by a medical professional and membership in one of the Christian denomination (orthodox, catholic, protestant, neo-protestant) or a general identification as Christian (e.g: “I’m a Christian and I was diagnosed with depression”, I’m Catholic/Orthodox/ Protestant and I was diagnosed with depression”). In addition, users had to be at least 18 years old and have an active account on the forums. Participants who reported other mental or physical disorders (e.g., schizophrenia, PTSD, OCD, and cancer) were excluded from the study. The discussion topics of the subreddits and other forums focused on Christianity and depression. Text searches were conducted using keywords, such as *depression*, *depressive disorder*, *diagnosed depression*, and *I have been diagnosed with depression*.

**Figure 1:**
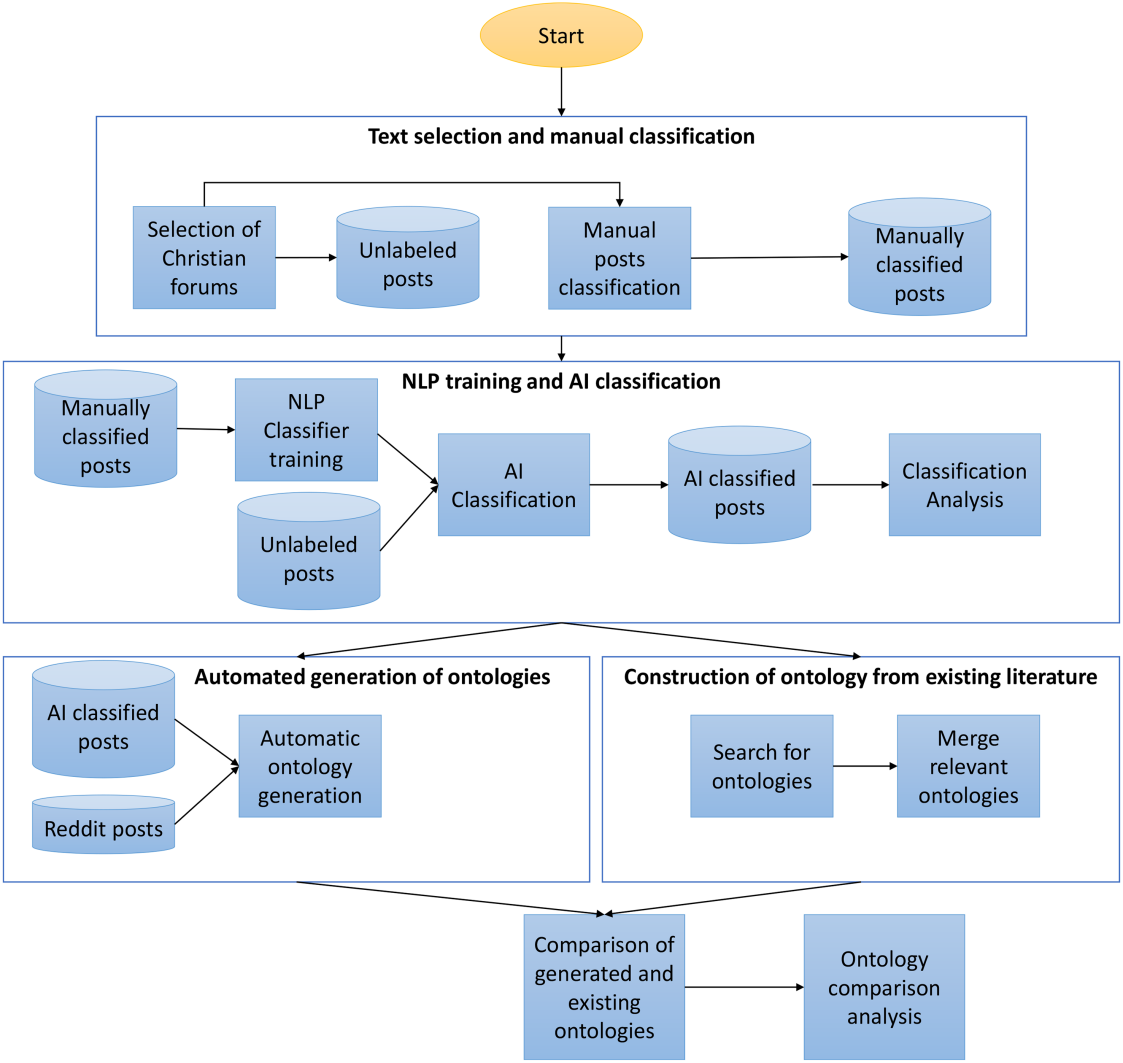
Research workflow.

A total of 93 posts were selected by psychologists on the research team and manually labeled. Of these, 36 posts were classified as belonging to Christians with depression, based on literature-supported criteria and explicit self-reports of having received a depression diagnosis from a medical professional. The remaining 57 posts formed the control group (General Christians) and did not include self-reports of a depression diagnosis. The posts had an average length of 313 words and were manually collected from publicly accessible forums associated with different Christian denominations (catholic, orthodox, and protestant). Figure 1 illustrates the research workflow.

The texts for the two categories—general depressed individuals who are not necessarily Christians (depressed) and general non-depressed individuals who are not necessarily Christians (non-depressed)—used to generate the respective ontologies were obtained from Reddit posts [Reddit Self-reported Depression Diagnosis, (RSDD), 2017].^1^ The data were obtained through a signed agreement between Georgetown University, USA and the University Politehnica of Bucharest, Romania. The posts for depressed individuals had a total number of words of approximately 195,538,000, while for general non-depressed individuals, it had a total number of words of approximately 733,020,000. As the tool for generating ontologies had some size limitations, we needed to cut the size of the text (more details in the section related to ontology generation). The site (see above) reports that the data were used in several other scientific research studies.

### 2.2 NLP training and AI classification

As mentioned in the introduction, to analyze texts and generate ontologies, we have created an NLP classifier for classifying texts from Christian forums. The classifier was trained on texts manually labeled by the psychologists on our team as authored by either depressed or non-depressed individuals, based on whether the user reported being diagnosed by a physician. The dataset consists of 93 posts, of which 36 belong to the depression group and 57 to the control group.

During preprocessing, we used regular expressions in Python to remove words containing digits or unknown characters and removed common English stop words. Each word in the posts was then lemmatized using the WordNetLemmatizer from the NLTK package. The post set was then vectorized using tf-idf, as machine learning algorithms require numeric input. The dataset was randomly split into training and testing sets using an 80–20 ratio.

First, we have evaluated several baseline classifiers on the training data using cross-validation and F1 as the scoring criterion. The results are presented in Table 1.

**Table 1:** Baseline classifier scores.

Classifier	F1 score	SD
Linear classifier with SGD training (SGD Classifier)	85%	0.11
Random forest classifier	82%	0.09
Logistic regression classifier	61%	0.18
Naive Bayes classifier for multinomial models	12%	0.15

The F1-score was selected as the primary evaluation metric because it provides a balanced measure that combines precision and recall. This metric is particularly appropriate for datasets with class imbalance, where accuracy alone may provide misleading results. In the context of depression detection, both precision (avoiding false positives) and recall (correctly identifying depressed cases) are important, making the F1-score a suitable and widely used performance measure.

The best results were obtained with the SGD classifier. Next, we have used a randomized search to select the best hyperparameters for SGD. We have set 2,000 iterations to select the loss, penalty, regularization rate, learning rate, class weight, and initial learning rate. The best score, based on F1-scoring, was 0.94 and obtained with the following hyperparameters:

- L2 penalty (standard SVM regularizer).
- hinge loss (linear SVM).
- invscaling learning rate.
- eta0 (initial learning rate) of 1.
- class weight of 60–40.
- learning rate of 0.01.

The classifier using these hyperparameters was then tested on the test data with the results shown in Table 2.

**Table 2:** Classification results.

Classifier	Precision	Recall	F1 score
SGD	83%	93%	86%

The trained classifier was then used to predict labels for unlabeled posts from Christian forums. From a total of 255 posts, 205 posts were classified as belonging to people with depression.

### 2.3 Automated generation of ontologies

The purpose of this research was to use automated methods to build, analyze, and then compare ontologies across four domains of interest: depressed Christians, non-depressed Christians, general non-depressed individuals (not necessarily Christians), and general depressed individuals (not necessarily Christians).

The generation of the concepts used in our ontologies was carried out in three steps:

Step 1: First, the corpus of text was built, as described above.

Step 2: Second, to extract relevant concepts from the corpora, we used the Text2Onto tool. This automated method is less time-consuming than manually extracting concepts from the text. An additional advantage of Text2Onto is its ability to assign a relevance score to each concept extracted from the corpus. The tool is capable of extracting concepts from large corpora of unstructured text.

While testing the concept extraction process using different corpora, we identified several limitations of Text2Onto. The most important limitation is related to the size of the corpus: the tool accepts a maximum of 90,000 characters, which is approximately 15,000 words. Corpora larger than this cannot be processed by Text2Onto. As a result, for each domain, we had to ensure that the corpora were below this size limit.

For the “general” cases, we were able to build larger corpora compared to the “Christian” cases. Since these two larger corpora exceeded the size limit, we divided them into smaller sub-corpora and ran the tool on each sub-part. The results were then combined to create a unified ontology for each domain (as described in Step 3).

By running the Text2Onto tool, we automatically generated a list of concepts for each domain of interest. For our research, we considered only the concepts with the highest relevance scores. Relevance is a positive value ranging from 0 to 1, and Text2Onto automatically sorts the generated concepts based on this score. In our ontologies, we included only concepts with a relevance score above a dynamically determined threshold. The threshold was computed based on the size of the corpus and the specific ontological domain. To evaluate the effect of the threshold, we built and analyzed domain ontologies using different concept limits: 100, 250, and 500.

Step 3: To build the final ontologies for the “general cases,” a parser was required. We implemented a parser in Java that processes the lists of concepts obtained from running Text2Onto on the sub-corpora generated in Step 2. The parser compares the concepts across these lists, and for each identical concept found, it computes the final relevance score as the sum of the relevance scores from each list.

**Figure 2:**
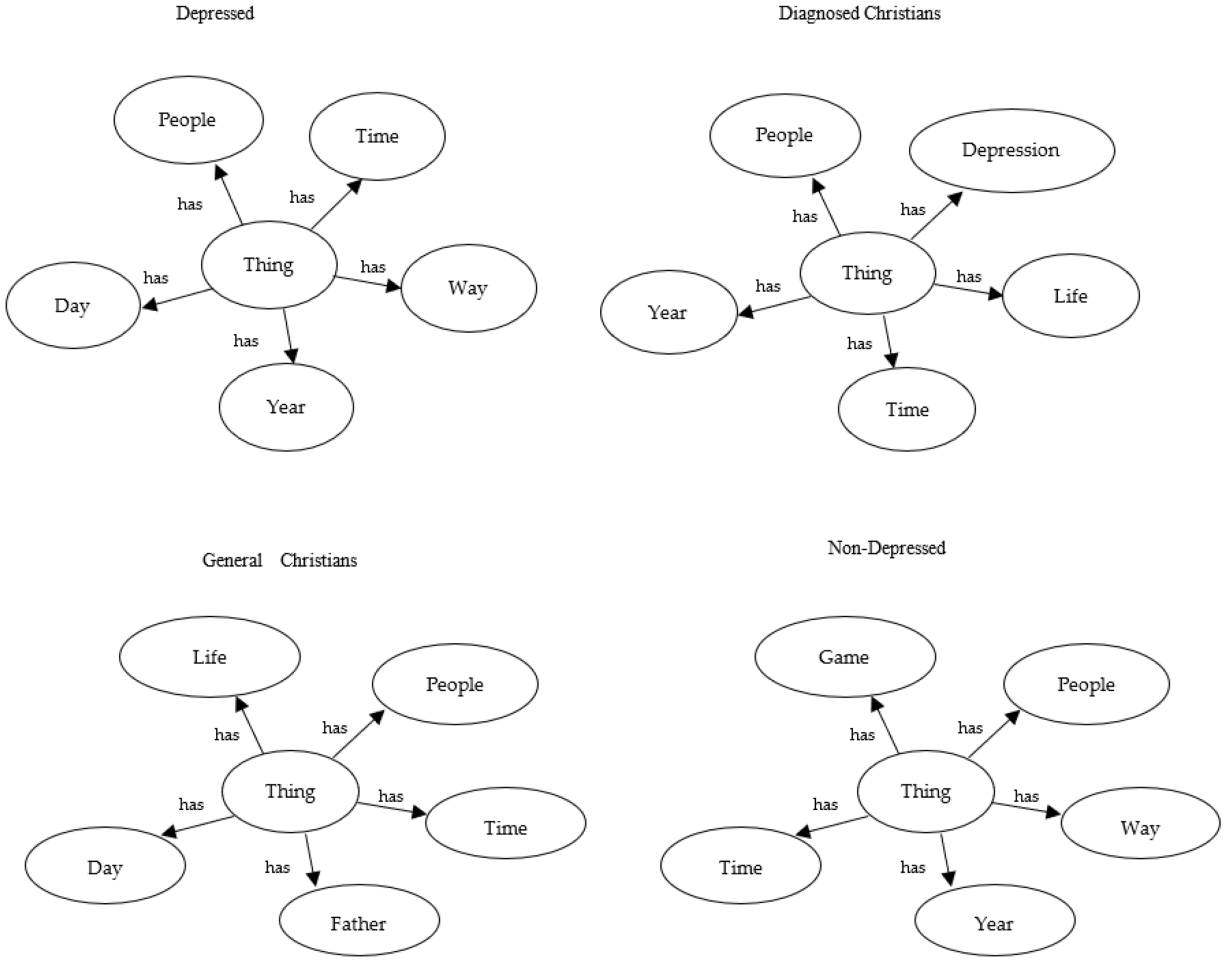
Sample main concepts for the generated ontologies.

A sample of the concepts used for each ontology can be analyzed (Figure 2).

### 2.4 Construction of ontology from existing literature

**Figure 3:**
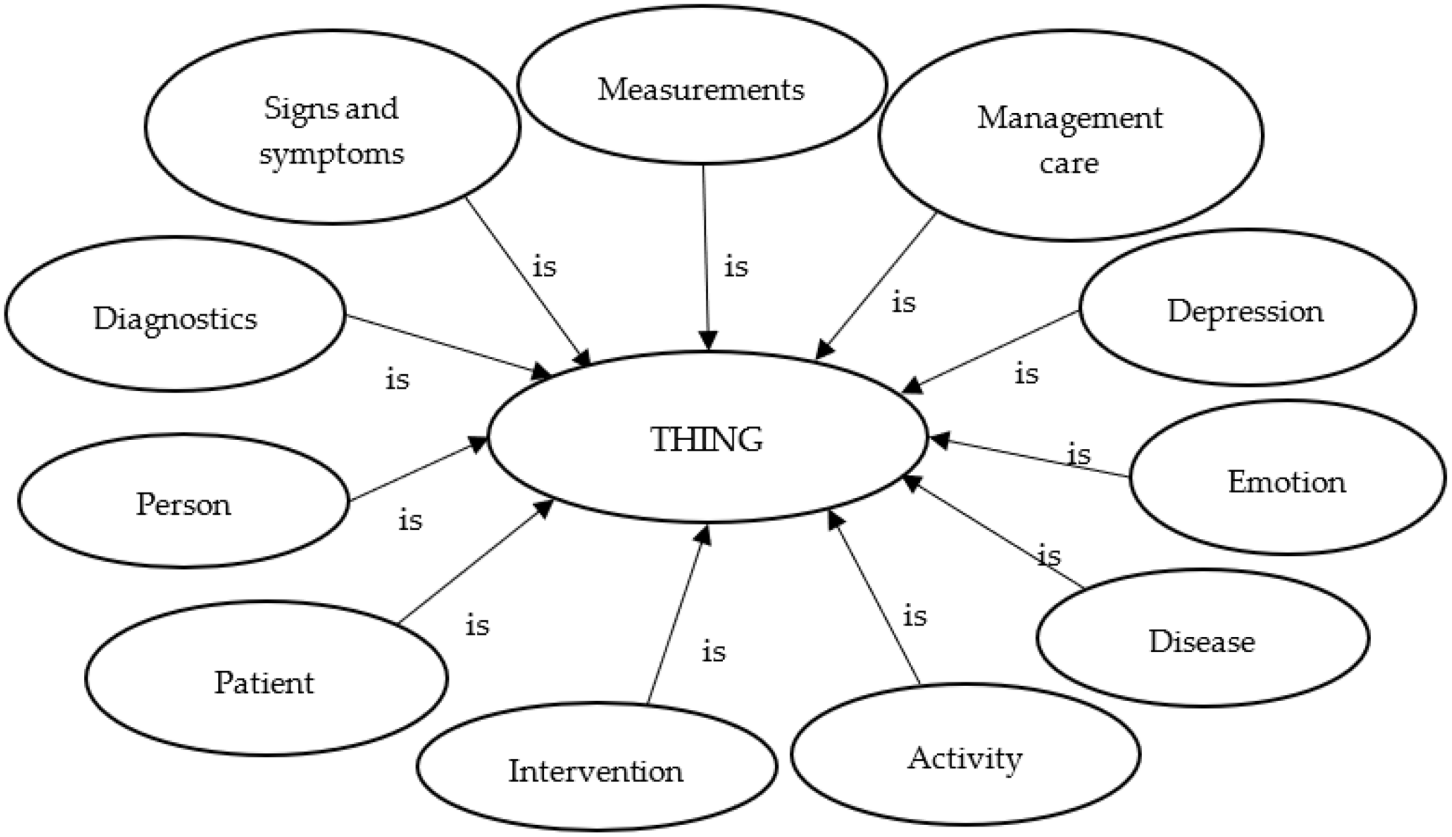
Root concepts in ontology from literature research.

In addition to the four categories of ontologies generated, we also built a literature-based ontology, which is described (Figure 3). We conducted a systematic search of the scientific literature using the following databases: Web of Science, Scopus, and Google Scholar. The string search used for the search was “ontology” AND “depress*.” Five relevant peer-reviewed articles published between 2015 and 2019 were identified. Three of the ontologies described depression in adolescents (Jung et al., 2015; Jung et al., 2016; Jung et al., 2017), while the remaining two focused on depression in the general population. Two ontologies aimed to provide a framework for applications designed to diagnose or assist in the treatment of depression (Chang et al., 2015; Petry et al., 2020), whereas the other three were intended to offer a semantic foundation for analyzing social media data.

Finally, only four of the five ontologies were found suitable for the purposes of our study, and only these ontologies were merged (Chang et al., 2015; Jung et al., 2015, 2016, 2017; Petry et al., 2020). One ontology was excluded (Jung et al., 2017) because it was a continuation of another study (Jung et al., 2016) and contained several terms that did not align with the framework. The selected ontologies were merged using the Protégé Web Ontology software.

We combined the main concepts found across all four ontologies, such as risk factors, signs, and symptoms of depression and intervention, as well as other concepts, such as measurement, email profile, and content posted online. Since each ontology used different words to classify these categories, we identified synonymous classes after importing the ontologies into Protégé Web Ontology. The resulting merged ontology comprised of 21 classes, 43 subclasses, and 58 individuals.

## 3 Results

To check the similarity of the generated ontologies, first, we computed their similarities according to the formula (Popa, 2020):

Similarity (Ont1, Ont2) = Nr-Com-Term/(Nr-terms-Ont1 + Nr-terms-Ont2).

Ontology similarity can be evaluated using several complementary components, such as concept similarity, hierarchical similarity, and relation similarity. Concept overlap represents a fundamental and widely used measure in ontology comparison. For example, Maedche and Staab (2002) define ontology similarity based in part on the overlap between concept sets, while Dellschaft and Staab (2006) evaluate automatically learned ontologies by comparing their concepts using shared-term-based precision and recall measures and extend this comparison to taxonomic hierarchies based on concept matching.

As the ontologies in our study were generated automatically using Text2Onto, which reliably extracts domain-relevant concepts but produces limited and less consistent relational structures, concept-based similarity provides the most robust and comparable measure of similarity between ontologies. This approach allows a consistent comparison of the semantic vocabularies extracted from the analyzed corpora.

We applied the formulas to our ontologies that had the concepts sorted according to the given relevance. As the generated ontologies had hundreds of terms, we decided to apply the formula to several dimensions of the ontologies, cutting them at 800, 500, and 200. The results are given in Tables 3–5.

**Table 3:** Similarity between ontologies limited to 800 concepts.

	Non-depressed	Diagnosed Christians	General Christians	Depressed
Non-depressed individuals	1.000	0.180	0.186	0.373
Diagnosed Christians	0.180	1.000	0.185	0.212
General Christians	0.186	0.185	1.000	0.206
Depressed individuals	0.373	0.212	0.206	1.000

**Table 4:** Similarity between ontologies limited to 500 concepts.

	Non-depressed	Diagnosed Christians	General Christians	Depressed
Non-depressed individuals	1.000	0.185	0.184	0.364
Diagnosed Christians	0.185	1.000	0.186	0.218
General Christians	0.184	0.186	1.000	0.215
Depressed individuals	0.364	0.218	0.215	1.000

**Table 5:** Similarity between ontologies limited to 200 concepts.

	Non-depressed	Diagnosed Christians	General Christians	Depressed
Non-depressed individuals	1.000	0.172	0.187	0.375
Diagnosed Christians	0.172	1.000	0.225	0.222
General Christians	0.187	0.225	1.000	0.222
Depressed individuals	0.375	0.222	0.222	1.000

It can be observed that there is no relevant difference between similarities at several lengths. Therefore, we can conclude that the reported similarity measures are robust. From the tables, we observe that the highest similarity ratio is between individuals in the general depressed group and those in the non-depressed groups, at approximately 0.37, while the lowest similarity is observed between Christians and non-Christians (both depressed and non-depressed), at approximately 0.18 for non-depressed individuals and 0.21 for general depressed individuals. Between the two categories of Christians, the similarity is approximately 0.18.

The highest degree of similarity between the two categories of non-Christians (0.37) may indicate that individuals in the general population share common experiences of sorrow, stress, and depression. In contrast, the similarity among religious individuals is lower, which may reflect the influence of their beliefs, potentially leading to differences in how depression is experienced across the two Christian categories.

In the next section, we compare the most relevant terms for the generated ontologies and the ones built from the literature.

Among general depressed and non-depressed individuals, although they represent distinct categories, many of the top 20 concepts are similar, including terms such as “people,” “game,” “year,” “shit,” and “someone.” Several conclusions can be drawn from these observations: games appear to be important for both groups, reflecting their relevance in contemporary life; relationships (e.g., “people” and “someone”) are significant; temporal references (e.g., “year” and “day”) may reflect daily stressors; and the use of curse words (e.g., “shit”) is relatively common.

Between the generated ontology for the depressed and the one built from literature, there are not many similar concepts. In literature-based ontology, concepts such as depression, emotions, and diagnosis are highly relevant, whereas they are less prominent in the ontology generated from texts. This discrepancy may be explained by the fact that individuals writing posts may be less severely depressed (as they have the energy and motivation to engage online) and may be less focused on the medical aspects of depression than experts anticipate. Both the similarity analyses and the examination of the most relevant concepts indicate that the contents produced by both depressed and non-depressed users share many common concepts, while showing less alignment with expert-derived perspectives.

Regarding the two categories of Christians, religious terms are prominently represented among the most relevant concepts, such as “God,” “faith,” “Christians,” and “church,” reflecting their religious beliefs. In depressed Christians, concepts such as “depression” and “anxiety” are more salient compared to non-depressed Christians, indicating that depression affects individuals regardless of their religious affiliation. For both Christian categories, social relationships (e.g., “people”) and temporal references (e.g., “year”) are also important concepts. In contrast, terms related to cursing or games are less prominent, indicating that they are not primary concerns for this population.

Two psychologists reviewed the classifier’s categorization of depressed and non-depressed Christians and validated its results. In the posts classified as belonging to depressed Christians, several depression-related symptoms were identified, such as feelings of sadness, impaired decision-making, physical symptoms, and a perceived need for help. The selected posts also emphasize the importance of recognizing the condition and seeking professional support.

Some posts discuss potential causes of depression, referring to mental, emotional, or physical factors, and describe personal experiences characterized by feelings of numbness or worthlessness. In this context, users also reflect on their experiences with mental health challenges, the role of medication, the importance of modifying maladaptive thought patterns, and the role of faith in their recovery process.

In addition, some posts address the misconception that Christians do not experience depression, along with the harmful comments individuals with depression may receive from members of their religious community, for example, being told that their depression is due to a lack of faith. Posts also reflect the belief that depression is a sign of weakness, leading to judgment and stigmatization of affected individuals. Such characteristics were considered by the psychologists when classifying posts as belonging to the depressed category.

Non-depressed Christians who report feelings of sadness or loneliness, emotions commonly associated with depression, or even those who mention having recently experienced depressive symptoms (without a confirmed diagnosis) display a distinct discourse pattern in which suffering is accepted as part of the human experience. Despite a sense of isolation being present, there remains a perceived sense of divine presence that mitigates the intensity of these emotions.

In this context, depression or suffering is often understood as being linked to personal choices, with individuals positioning themselves as active agents capable of influencing their emotional states, a perspective that contrasts with the helplessness typically associated with clinical depression. Furthermore, even when describing a level of suffering that is difficult to endure, these individuals explicitly state that suicide has never been considered an option.

Such characteristics were taken into account by the psychologists when classifying posts as belonging to the non-depressed category.

## 4 Discussion and conclusion

The purpose of this research was to use automated methods to build, analyze, and compare ontologies for four categories, which refer to depressed Christians, non-depressed Christians, general non-depressed individuals (not necessarily Christian), and general depressed individuals (not necessarily Christians). To the best of our knowledge, this is the first study to address this topic within a religious population.

The results indicated no substantial differences in similarity across the four categories. Another objective of the study was to apply automatic ontology generation to a corpus of social media posts and to conduct both qualitative and quantitative comparisons among the four categories. Regarding the most frequently occurring terms in posts authored by depressed individuals, our findings are consistent with those reported by Mustafa et al. (2020), who identified similar keyword weights for depression classification. This convergence supports the potential generalizability of our results.

Regarding the differences between religious individuals diagnosed with depression and those in the general depressed group, the classification based on word usage indicates that the religious dimension is particularly salient for the religious group. This finding is consistent with previous studies that have examined the role of religion as a coping mechanism, suggesting that religious beliefs may serve as a resource in managing depression (Dolcos et al., 2021).

One limitation of the study is the relatively small sample size, particularly for the texts selected from Christian participants, which were drawn from a limited number of posts compared to those from non-Christian individuals with depression. Another limitation concerns the generalizability of the NLP classifier. The classifier was trained on Reddit and Christian forum posts, which share similar characteristics, such as informal language, personal narratives, and discussions of mental health. However, linguistic patterns, user demographics, and communication styles may differ across other platforms or populations. Therefore, the classifier’s performance may not fully generalize to different contexts, such as clinical texts or other social media platforms. Future research should validate and refine the classifier using more diverse datasets to improve its generalizability.

Another methodological limitation is the use of our own classifier for the text-to-ontology analysis. Although this tool has limitations in identifying relationships between concepts, the comparisons conducted in this study were based primarily on the extracted concepts rather than on the connections among them. Therefore, this limitation is unlikely to have significantly influenced the results.

As a future direction, such a system could be integrated into a counseling platform to facilitate early detection of depression, as early diagnosis may support the timely implementation of effective intervention strategies.

## Data Availability

The data analyzed in this study is subject to the following licenses/restrictions: The data presented in this study are available upon request form the corresponding author. Requests to access these datasets should be directed to: NG, n.goga@rug.nl.

## Footnotes

1
https://georgetown-ir-lab.github.io/emnlp17-depression/
